# Analysis of risk factors for mild cognitive impairment based on word list memory test results and questionnaire responses in healthy Japanese individuals registered in an online database

**DOI:** 10.1371/journal.pone.0197466

**Published:** 2018-05-17

**Authors:** Masayo Ogawa, Daichi Sone, Kazushi Maruo, Hiroyuki Shimada, Keisuke Suzuki, Hiroshi Watanabe, Hiroshi Matsuda, Hidehiro Mizusawa

**Affiliations:** 1 Integrative Brain Imaging Center, National Center of Neurology and Psychiatry, Tokyo, Japan; 2 Translational Medical Center, National Center of Neurology and Psychiatry, Tokyo, Japan; 3 University of Tsukuba, Tsukuba, Japan; 4 Center for Gerontology and Social Science, National Center for Geriatrics and Gerontology, Aichi, Japan; 5 Innovation Center for Clinical Research, National Center for Geriatrics and Gerontology, Aichi, Japan; 6 National Center of Neurology and Psychiatry, Tokyo, Japan; University of Kentucky, UNITED STATES

## Abstract

Although the development of effective therapeutic drugs and radical treatment options for dementia and Alzheimer’s disease (AD) remains urgent, progress in recent clinical trials of AD drugs has been less than adequate. In order to advance the progress of clinical trials, it is necessary to establish more efficient methods of recruitment. In Japan, there are registration systems stratified by mild cognitive impairment and preclinical and clinical stages of early and advanced stage dementia, but there is no registration system for healthy individuals yet. Therefore, in the present study, we developed a large-scale, internet-based health registry to investigate factors associated with cognitive function among registered participants. A total of 1038 participants completed the initial questionnaire and word list memory test. Among these participants, 353 individuals completed a second questionnaire and memory test. Stepwise multiple regression analysis was performed using IBM SPSS version 23.0 for Windows at a statistical significance level of p<0.05. We found that mood, motivation, and a decreased ability to perform activities of daily living were significantly associated with cognitive function. The results of the present study suggest that maintaining social involvement is important to prevent decreases in physical activity, daily function, mood, and motivation.

## Introduction

The number of people with dementia in Japan is estimated to reach 7 million by the year 2025 [1]. Despite the urgent need to develop therapeutic strategies for dementia and Alzheimer’s disease (AD), progress in recent clinical trials of AD drugs has been less than adequate [2]. Matsuda et al. reported that over the past 10 years, clinical trials for AD modifying therapies have been largely unsuccessful, partly due to difficulties in recruiting early stage patients for enrollment [3]. Until therapeutic drugs are developed, it is essential to examine risk factors for dementia and intervene in lifestyle habits that may put one at a risk of AD. A recent review reported that modifiable risk factors for AD are mostly related to either cardiovascular risk factors (diabetes, hypertension, and obesity) or lifestyle habits (e.g., smoking, physical activity, diet, and mental and social activity) [2]. Thus, until more effective therapeutic drugs and radical treatment options are developed for AD, the most promising strategies require the assessment and modification of risk factors for dementia. Although the development of radical therapeutic strategies remains critical, the importance of creating a registry system for individuals with normal cognitive function at a risk of AD cannot be understated. To ensure that clinical trials targeting mild cognitive impairment (MCI) and the preclinical/early stages of AD are conducted efficiently, it is necessary to establish more appropriate methods for large-scale clinical trial recruitment [4]. In clinical research aimed at preventing dementia, a large-scale registration system is necessary to make it scale to validate its efficacy. The American Global Alzheimer's Platform [5] and European Prevention of Alzheimer's Dementia [6] were designed to develop new treatments for secondary prevention. These are actively thriving internet-based registries. In Japan, however, there is not yet a large-scale registration system. It is necessary to recruit enough participants to validate its efficacy. Therefore, it is necessary to establish a system aiming at facilitating patient registration in clinical trials and, at the same time, use this registration system as a platform for preventive clinical research.

Thus, in the present study, we aimed to create a large-scale, internet-based registry system for healthy people, known as the Integrated Registry of Orange Plan (IROOP^®^), to identify not only factors associated with cognitive function, but also those which affect changes in cognitive function over a 6-month period.

## Participants and methods

### Participants

Registration in the IROOP^®^ system began on July 5, 2016. The present study included 1038 individuals whose registration information, responses to all items of the initial questionnaire, and word list memory test results (MCI Screen) were entered on or before August 15, 2017 (mean age: 59.0±10.4 years; 400 men and 638 women; Table 1), as well as 353 individuals who had completed the follow-up questionnaire and a second MCI Screen 6 months after completing the initial questionnaire (mean age: 60.2± 10.0 years; 139 men and 214 women; Table 2).

**Table 1:** Summary of subjects who completed the initial questionnaire and MCI Screen (n = 1038).

	Age groups						
	all	40–49	50–59	60–69	70–79	80–89	*p* value
n	1038	224	331	314	139	30	
	mean±S.D.	mean±S.D.	mean±S.D.	mean±S.D.	mean±S.D.	mean±S.D.	
age	59.0±10.4	45.5±2.8	54.6±2.8	64.6±2.8	74.0±2.6	81.8±1.9	.000
m:f	400:638	61:163	85:246	141:173	93:46	20:10	.000
Education years	14.8±2.2	15.2±2.3	15.0±2.1	14.8±2.2	14.2±2.4	14.2±2.1	.000
MPI score	69.6±9.4	77.5±5.9	73.3±6.0	66.0±6.9	59.5±8.4	52.8±6.1	.000

MCI Screen, mild cognitive impairment screen (word list memory test)

S.D., standard deviation;m, male; f, female; MPI, memory performance index.

**Table 2:** Summary of subjects who completed the second questionnaire and MCI Screen (n = 353).

	Age groups						
	all	40–49	50–59	60–69	70–79	80–89	*p* value
n	353	60	114	120	50	9	
	mean±S.D.	mean±S.D.	mean±S.D.	mean±S.D.	mean±S.D.	mean±S.D.	
age	60.2±10.0	45.9±2.8	54.8±3.1	67.1±7.2	74.1±2.5	82.0±1.7	.000
m:f	139:214	22:38	19:95	58:62	34:16	6:3	.000
Education years	14.9±2.1	15.1±2.4	14.7±1.9	14.9±2.0	14.9±2.3	14.9±2.3	.879
Second MPI score	70.3±9.3	79.3±5.3	74.3±5.6	67.0±7.2	61.7±7.4	50.2±10.2	.000
First MPI score	70.0±8.8	78.1±5.9	74.0±5.7	67.1±6.8	60.7±8.1	55.8±5.4	.000

MCI Screen, mild cognitive impairment screen (word list memory test)

S.D., standard deviation; m, male; f, female; MPI, memory performance index.

This internet-based registry system targeted healthy Japanese people whose cognitive function has not remarkably deteriorated and were aged 40 years or older, living in Japan, and native Japanese speakers. This study excluded those who had already been diagnosed with AD, MCI, dementia, frontotemporal dementia, Lewy body type dementia, psychiatric disorders (major depression, bipolar disorder, anxiety disorder, obsessive compulsive disorder, posttraumatic stress disorder, panic disorder, schizophrenia, and eating disorders), and those taking symptom-controlling drugs, such as Aircept. We recruited participants using advertising media, such as the television and newspaper. In addition, we explained our registry and recruited participants at a lecture open to the public and research societies.

### Internet-based questionnaire items

Using the questionnaire administered to patients in the Brain Health Registry (http://www.brainhealthregistry.org/) of the United States as a reference, although there are differences in the number depending on participants, we generated approximately 220 items for the online questionnaire. After logging into the IROOP^®^ system (https://www.iroop.jp/), participants entered their consent and registration information, following which personal pages were created (“My Pages”). Participants accessed the initial questionnaire on their respective My Pages. The follow-up questionnaire was displayed on the My Page 6 months after the initial questionnaire had been completed. Questionnaire items were categorized as follows:

- Items required for registration: e-mail address, birthday, gender, years of education, race, prefecture of residence, and the presence or absence of any housemates.
- Items on the initial questionnaire: weight, height, lifestyle (demographics, mood, quality of life, sleep patterns, and diet), and medical history (present illness, medication, past history, family history, past history of head and neck injuries or concussion, and daily cognitive function).
- Items on the follow-up questionnaire (administered every 6 months; note that registration in this system is in progress and this study uses the initial questionnaire and the first six month follow up data of the first time and the first regular time in this study): health status, mood, quality of life, sleep patterns, diet, medication history, history of present illness, past history of head and neck injuries and concussion, and daily cognitive function.

### Questionnaire responses

The questionnaires included items that required yes/no responses, as well as those for which multiple response options were provided. For example, participants chose either “1: yes” or “2: no” when presented with the question, “Do you feel that your life is empty?” For questions with multiple response options, participants rated their responses using a scale similar to the following: “1: very difficult,” “2: a little difficult,” or “3: not difficult at all.” The questionnaire also included open-ended items for which participants could provide unique responses.

### Word list memory test

Whenever participants completed either the initial or periodic questionnaire, a toll-free telephone number was posted on their My Page. Calling the number enabled participants to take the Japanese version of the word list memory test (MCI Screen) [7] for free. The MCI Screen is a simplified scale for the assessment of cognitive function that has been approved by the United States Food and Drug Administration. The examination takes approximately 15 minutes to complete and the questions differ for each session. A scoring algorithm is then used to calculate a memory performance index (MPI) score based on the patient’s test results, age, educational background, and race [8]. The MPI quantifies the pattern of correctly recalled words from the Consortium to Establish a Registry for Alzheimer’s Disease wordlist on a scale from 0 to 100, which distinguishes normal from MCI with an accuracy rate of 96–97% [8].

### Statistical analysis

Statistical analyses were performed using IBM SPSS version 23.0 for Windows (SPSS Inc., Tokyo, Japan). Differences in age groups were analyzed using an ANOVA and χ^2^ tests were used to analyze differences in categorical variables. Stepwise multiple regression analyses were used to identify questionnaire items associated with the MPI score, using the initial MPI score as the dependent variable and each questionnaire item as the independent variable. In the present study, nonessential questionnaire items and open-ended questions were excluded from analysis. Moreover, items that were irrelevant to all participants in the initial questionnaire (previous history of non-parkinsonian motor disorders, Huntington’s disease, amyotrophic lateral sclerosis, motor neuron diseases other than amyotrophic lateral sclerosis, and multiple sclerosis) were excluded from analyses.

Next, we performed stepwise multiple regression analysis to identify which questionnaire items were associated with longitudinal changes in cognitive function, using the difference between the initial and follow-up MPI scores as the dependent variable and each questionnaire item as the independent variable. These analyses were performed using data from the 353 participants who completed the follow-up questionnaire and the second MCI Screen 6 months after completing the initial questionnaire. Similarly, items that were irrelevant to all participants (history of Parkinson’s disease, non-parkinsonian motor disorders, Huntington’s disease, amyotrophic lateral sclerosis, motor neuron diseases other than amyotrophic lateral sclerosis, multiple sclerosis, medication history, obsessive compulsive disorder, hoarding disorder, and mental disorder) were excluded from analyses. The level of statistical significance was set at p<0.05.

### Ethical considerations

The present study was approved by the ethics committee of the National Center of Neurology and Psychiatry. All included participants provided informed consent by marking the appropriate box on the IROOP^®^ home page, on which the full details of the study were posted. This study was registered in the University Hospital Medical Information Network Clinical Trials Registry (UMIN000022795).

## Results

### Participant characteristics

Table 1 presents the characteristics of the 1038 participants who had completed the initial questionnaire and the MCI Screen. Among participants in their 40s to 60s, the proportion of women was greater than that of men, whereas the reverse was true among participants in their 70s and 80s We observed no significant differences in the years of education among the age groups, likely due to the use of an internet-based registry system. MPI scores decreased along with increases in age.

Table 2 presents the characteristics of the 353 participants who completed the follow-up questionnaire and second MCI Screen. Again, the proportion of women was greater than that of men among participants in their 40s to 60s, whereas the reverse was true among participants in their 70s and 80s. MPI scores also decreased along with increases in age. There was no significant difference in MPI score and gender among age groups; however, there was a significant difference in the years of education.

### Multiple regression analysis of each questionnaire item and MPI scores

Table 3 shows the results of the stepwise multiple regression analysis of the initial MPI scores, including the coefficients linking each independent variable to the dependent variable.

**Table 3:** Stepwise multiple regression analysis results with initial score as dependent variable.

	Estimates of beta coefficient	SE	*t value*	*p value*	
Age (age at the end of questionnaire response)	-.566	.020	-27.998	.000	**
Gender	3.625	.411	8.819	.000	**
Education years	.252	.087	2.895	.004	**
Dose your health now limit you in these activities? Bathing or dressing yourself	6.893	1.824	3.780	.000	**
Compared to 10 years ago, has there been any change in Executive Functioning Planning -Developing a schedule in advance of anticipated events	-2.259	.640	-3.530	.000	**
Were you diagnosed with motor development delay (e.g., late walking or difficulties with fine movements/ learning to use tools)?	10.156	3.455	2.940	.003	**
Compared to 10 years ago, has there been any change in executive functioning: organization—Using an organized strategy to manage a medication schedule involving multiple medications	1.107	.397	2.787	.005	**
Do you feel happy most of time?	-1.463	.587	-2.492	.013	*
During the past month, how would you rate your sleep quality overall?	.978	.349	2.804	.005	**
Compared to 10 years ago, has there been any change in…Executive functioning Divided Attention—Cooking or working and talking at the same time	-.907	.365	-2.482	.013	*
How many times a week do you eat chicken ?	.219	.101	2.169	.030	*
Do you currently have or have had any of the following conditions in the past?—Cancer-	1.557	.690	2.256	.024	*
Do you participate in events at the public hall ?	1.045	.434	2.408	.016	*
Do you currently have or have had any of the following conditions in the past?—Diabetes -	1.978	.889	2.224	.026	*
During the past 4 weeks, how much did pain interfere with your normal work (including both work outside the home and housework)?	.778	.273	2.849	.004	**
During the past month, how much of a problem has it been for you to keep up enough enthusiasm to get things done?	-.760	.299	-2.537	.011	*
Compared to 10 years ago, has there been any change in Language–understanding the point of what other people are trying to say	1.036	.391	2.649	.008	**
Do you usually play with your head or do chess?	-.947	.419	-2.262	.024	*
How many snacks do you eat each day?	.504	.245	2.058	.040	*
Adjusted *R*^2^	.601				
n	1038				

*p < .05

**p < .01

MPI scores were significantly associated with the following questionnaire items: age, gender, years of education, the extent of changes in the ability to adjust one’s schedule in advance for anticipated events over a 10-year period; the extent of difficulty in bathing and dressing alone, and a past history of cancer or diabetes mellitus. The coefficient of determination for the generated model (*R*^*2*^) was 0.608 (*p<*0.05). Although MPI scores decreased along with increases in age, these scores were significantly higher among women than men.

We then examined the answers for each questionnaire item by MPI score. For the question, “how difficult is it for you to bathe and dress by yourself, according to your health status?,” participants chose the most appropriate response from among the following three options: “1: very difficult,” “2: a little difficult,” or “3: not difficult at all.” The MPI scores of participants who responded with “3: not difficult at all” were approximately 3.8 points higher than those who responded with “1: very difficult.” For the question, “during the past month, how much of a problem has it been for you to have enough enthusiasm to get things done?,” participants chose the most appropriate response from among the following four options: “1: no problem at all,” “2: only a little problem,” “3: some problem,” and “4: considerable problem.” The MPI scores of participants who responded with “3: considerable problem” were approximately 2.5 points lower than those who responded with “1: no problem at all.” For the question, “do you currently have cancer or have you ever had cancer?,” participants chose either “1: yes” or “2: no.” The MPI scores of participants who responded with “2: no” were approximately 2.3 points higher than those of participants who responded with “1: yes.” For each additional year of education, MPI scores increased by approximately 2.9 points.

Among the items shown in Table 3, the following factors were most strongly associated with MPI score: age, gender, difficulty in bathing and dressing alone according to health status, difficulty in maintaining enthusiasm for accomplishing tasks, and ability to create a schedule before an expected event.

Multiple regression analysis was then used to evaluate data from the 353 participants who completed the baseline and follow-up questionnaires as well as the MCI Screen (Table 4).

**Table 4:** Stepwise multiple regression analysis using the difference between the first and second scores as dependent variables.

	Estimates of bete coefficient	SE	*t value*	*p value*	* *
MPI Score (first time)	-.469	.040	-11.711	.000	**
Age (age at the end of questionnaire response)	-.347	.036	-9.678	.000	**
Do you currently have or have had any of the following conditions in the past?—Traumatic Brain Injury -	12.264	3.295	3.722	.000	**
Do you currently have a cardiac pacemaker or defibrillator in your body?	-15.815	4.554	-3.473	.001	**
How many times a week do you eat dinner away from home?	-.781	.267	-2.922	.004	**
Compared to 6 months ago, are you pursuing fewer or more activities and interests?	1.983	.529	3.745	.000	**
Do you currently have any surgical metal or any foreign objects in your body?	2.516	1.046	2.405	.017	*
Do you feel that your life is empty?	3.358	1.093	3.071	.002	**
Compared to 10 years ago, has there been any change in executive functioning: organization—Keeping mail and papers organized	1.004	.422	2.378	.018	*
Have you ever been diagnosed with any of the following psychiatric conditions? -Essential tremor-	8.737	4.555	1.918	.056	
During the past 4 weeks, to what extent has your physical health or emotional problems interfered with your normal social activities with family, friends, neighbors, or groups?	-1.853	.507	-3.653	.000	**
Do you feel that your situation is hopeless?	-1.490	.538	-2.773	.006	**
Do you currently have or have had any of the following conditions in the past?—Hearing Loss -	5.472	2.312	2.366	.019	*
During the past 4 weeks, have you had any of the following problems with your work or other regular daily activities as a result of any emotional problems(such as feeling depressed or anxious) Accomplished less than you would like ?	-2.091	.730	-2.865	.004	**
During the past month, how often have you had trouble sleeping because you–Have pain -	-1.335	.399	-3.344	.001	**
Is chronic pain a problem for you?	-1.528	.565	-2.703	.007	**
Compared to 10 years ago, has there been any change in…Visual-spatial and perceptual abilities—Finding way around a house visited many times	-1.886	.839	-2.248	.025	*
Do you currently smoke tobacco or have smoked tobacco in the past?	1.344	.551	2.440	.015	*
During the past month, how long(in minutes) has it usually taken you to fall asleep each night?	-.261	.124	-2.097	.037	*
During the past month, how often have you had trouble sleeping because you—Had bad dreams?	.824	.410	2.009	.045	*
Adjusted *R*^2^	.413				
n	353				

*p < .05

**p < .01

These analyses revealed that the following questionnaire items were associated with changes in MPI scores during the 6 months between the initial and follow-up examinations: initial MPI score, age, feelings of emptiness, an increase or decrease in daily activities or interests over the past 6 months, among others. The coefficients representing the contribution of each independent variable to the dependent variable are shown in Table 4. The following questionnaire items, listed in descending order of *t* value, were associated with changes between the initial and second MPI scores: initial MPI score, age, an increase or decrease in daily activities or interests over the past 6 months, and a past history of traumatic brain injury.

When asked to evaluate changes in the ability to organize things (e.g., mail, papers, etc.) over the past 10 years, participants selected their responses from among the following five options: “1: better than before or unchanged,” “2: questionable/sometimes worse,” “2.5: unknown,” “3: gradually worsening,” and “4: increasingly worsening.” The MPI scores of participants who selected “1: better than before or unchanged” were approximately 2.4 points higher than the scores of those who selected “4: increasingly worsening.” Furthermore, the MPI scores of participants who reported no history of hearing loss were approximately 2.4 points higher than those who reported such a history. The MPI scores of participants who reported feeling that their situation was “hopeless” were approximately 2.8 points lower than those who did not report such feelings.

## Discussion

In the present study, we aimed to identify factors associated with cognitive function (MPI score) as well as those affecting changes in cognitive function over a 6-month period among healthy Japanese adults registered in the IROOP^®^ database. Stepwise multiple regression analysis revealed that 19 factors from the initial questionnaire and 20 factors from the follow-up questionnaire were significantly associated with MPI score. Factors significantly associated with initial MPI score included difficulty bathing/dressing alone, level of happiness most of the time, history of cancer, the extent of change in the ability to make adjustments to one’s schedule in advance, and history of diabetes mellitus (Table 3). Multiple regression analysis of data from 353 participants revealed that the following items were significantly associated with changes between the initial and follow-up MPI scores: Compared to 6 months ago, are you pursuing fewer or more activities and interests? Do you feel that your life is empty? and feelings of hopelessness regarding one’s general situation.

In the present study, participants were asked to evaluate their extent of difficulty in bathing and dressing alone. This item, which was significantly associated with initial MPI score, reflects basic activities of daily living that involve physical movements. Fratiglioni et al. [9] highlighted the destructive nature of confining oneself to the home in old age, as it leads to decreased physical activity and human interaction that contributes to the rapid deterioration of mental and physical function. Sabia et al. [10] reported that a lower risk of dementia in physically active people may be attributed to reverse causation; that is, due to a decline in physical activity levels in the preclinical phase of dementia.

Such impairments; MCI then lead to further reductions in activity, allowing the cycle to continue. Thus, regular physical exercise and maintaining the ability to perform basic activities of daily living should be promoted, as these may aid older adults in preventing the development and progression of substantial MCI.

Moreover, our analysis of initial responses revealed that the MPI scores of participants who reported feeling happy most of the time were approximately 2.5 points higher than those of participants who responded otherwise. In addition, the MPI scores of participants who reported no difficulty in maintaining enthusiasm for accomplishing tasks were approximately 2.5 points higher than those who reported considerable difficulty in maintaining enthusiasm. A constant state of low mood or enthusiasm causes depressive symptoms, which have been identified as risk factors for social isolation and reduced activity [11]. In contrast, social involvement, intellectual activities, and social networks have been recognized as protective factors against the development of dementia [12]. The magnitude of the association of social participation is comparable to other well-established predictors of cognitive functioning, providing evidence that social participation plays an important role in cognitive functioning and successful aging [13]. To prevent decreases in physical activity and human interaction due to a rapid progression of physical and cognitive impairment, clinicians and researchers should stress the importance of social integration and maintaining the ability to perform activities of daily living.

Given these findings, the availability of social opportunities and activities outside the home for middle-aged and older adults seems to be critical for maintaining physical and cognitive function. In Japan, a social project called the Dementia (Orange) Café has been implemented in various communities, medical institutions, and other venues. This is one of the main policies presented in the Comprehensive Strategy to Accelerate Dementia Measures (New Orange Plan) issued by the Ministry of Health, Labour, and Welfare. At these cafés, anyone, including those diagnosed with dementia and their families, as well as others interested in the prevention of dementia, can connect with local communities and specialists in their region. The results of the present study highlight the need for public agencies and organizations to support this and similar projects.

Previous prospective cohort studies have revealed that lifestyle-related diseases and lifestyle factors are closely associated with the incidence of AD [9]. Consistent with the 1988 Hisayama Study that demonstrated that the incidence of AD was significantly higher among patients with diabetes mellitus [14], our findings suggest that diabetes mellitus is associated with cognitive impairment. Likewise, the Hisayama Study reported smoking as a risk factor, which is consistent with our findings [15]. Although the present study yielded some results that were comparable to those of previous studies, our findings also indicated that MPI scores were higher in participants who reported poor sleep quality and poor participation in events at the public hall. As these results are opposed to those of previous studies, they must be interpreted with caution. Future studies should investigate the relevance of these factors in AD among a larger group of participants over a longer period of time.

The level of education did not significantly differ among age groups for participants who completed the follow-up questionnaire. Such findings suggest that highly educated older adults are likely to be interested in studies that rely on this type of registry system.

As less than half of the number of people who completed the initial questionnaire also completed the follow-up assessment, there is a possibility that the low response rate generated response bias. People who are confident in their memory may have completed the follow-up assessment. It is necessary to keep in mind the possibility that someone who lacks confidence in their memory did not complete the follow-up assessment. This represents a potential limitation of our study, in that our findings may not be generalizable to older adults with lower levels of education.

Among the 353 participants who completed MPI assessments at the 6-month follow-up, some individuals exhibited improvements in cognitive function between the first and second assessments. This finding indicates that those who were interested in this registry system and participated in this study may also have been interested in the prevention of dementia and managing modifiable risk factors for dementia in their daily lives. In addition, although the questions in the MCI Screen differed for each session, we speculate that participants may have become more familiar with taking the test over the telephone by the second session. Indeed, previous neuropsychological studies have reported that responses to repeated stimulation follow a natural course, eventually reaching a plateau [16]. Thus, the effect of increasing familiarity to the assessment may subside with more follow-up sessions.

The items of traumatic brain injury have been extracted this time, and it is necessary to carefully follow people who have this clinical history. In addition, there are items related to pain as the first extracted as common in both the first and second times, and it also shows the importance of first removing organic pain mechanically. Secondly, there were items related to mood and motivation; it will be necessary to identify old age depression, as this could lead to early interventions for depression.

At the 2017 International Conference of Alzheimer’s Disease International in London, the following risk factors for dementia were among those identified as modifiable factors: depression, obesity, diabetes mellitus, decreased social interaction, and lack of exercise [17]. Because numerous studies have also reported relatively consistent results regarding risk and preventive factors for dementia [2], a consensus has begun to emerge. Indeed, the present study identified certain activities of daily living and diabetes mellitus as risk factors for dementia. Because the currently available drug therapies prevent the progression of dementia only to a limited extent, efforts should be made to prevent or delay the onset of dementia via the modification of significant risk factors. We aim to continue our investigation of modifiable factors in future studies.
